# Supplementary material for: Key Regulators of Angiogenesis and Inflammation Are Dysregulated in Patients with Varicose Veins
Source: Int J Mol Sci. 2024 Jun 20;25(12):6785. doi: 10.3390/ijms25126785 (PMC11204110; doi:10.3390/ijms25126785)
Supplement: Supplementary file 1 [file ijms-25-06785-s001.zip › Supplementary_Figures_submission.pdf]

SUPPLEMENTARY FIGURES

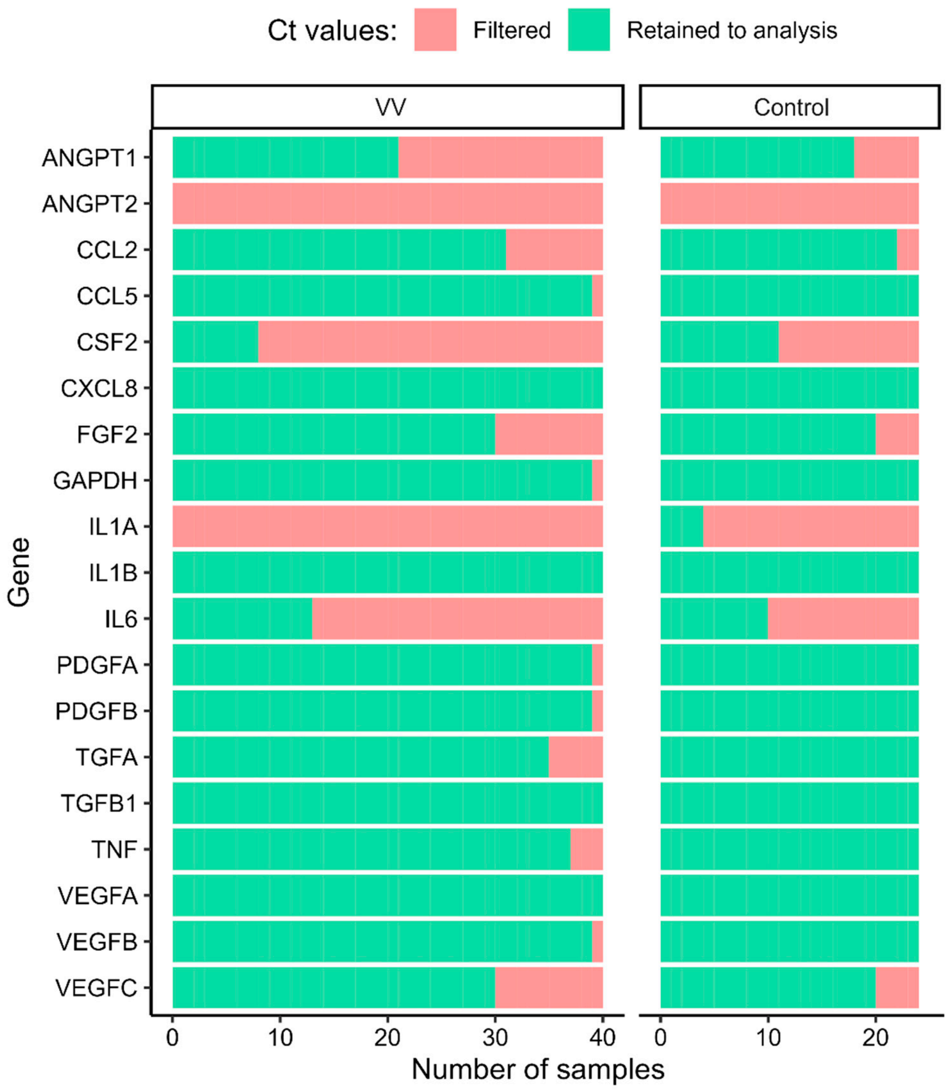

Figure S1. The amounts of samples with filtered and retained Ct values.

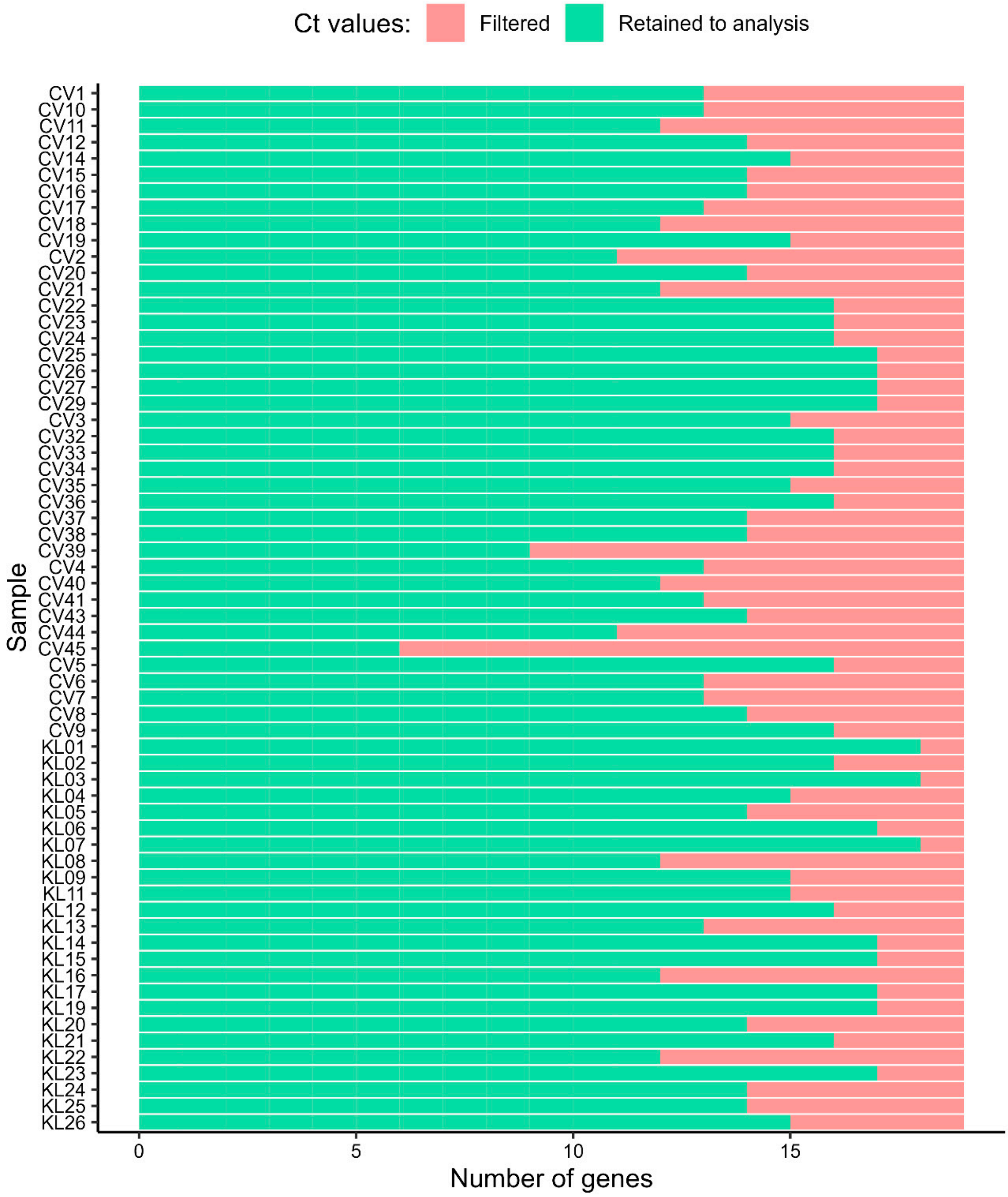

Figure S2. The amounts of genes with filtered and retained Ct values in analyzed samples.

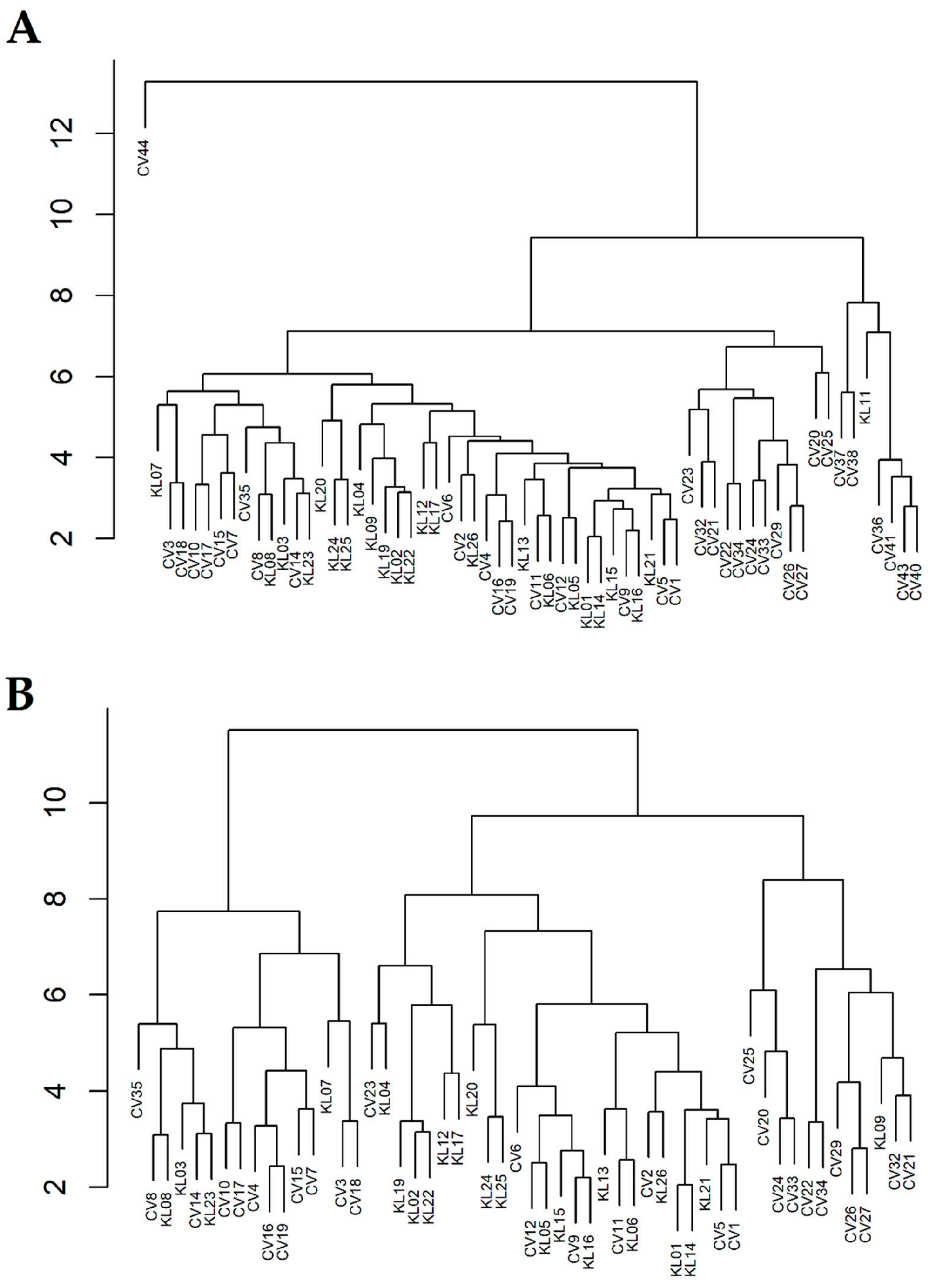

Figure S3. Hierarchical analysis performed for gene expression data (2^{-dCt} values) of the analyzed samples using Canberra distances. (A) Results obtained before removing outlier samles and (B) after removing outlier samples.

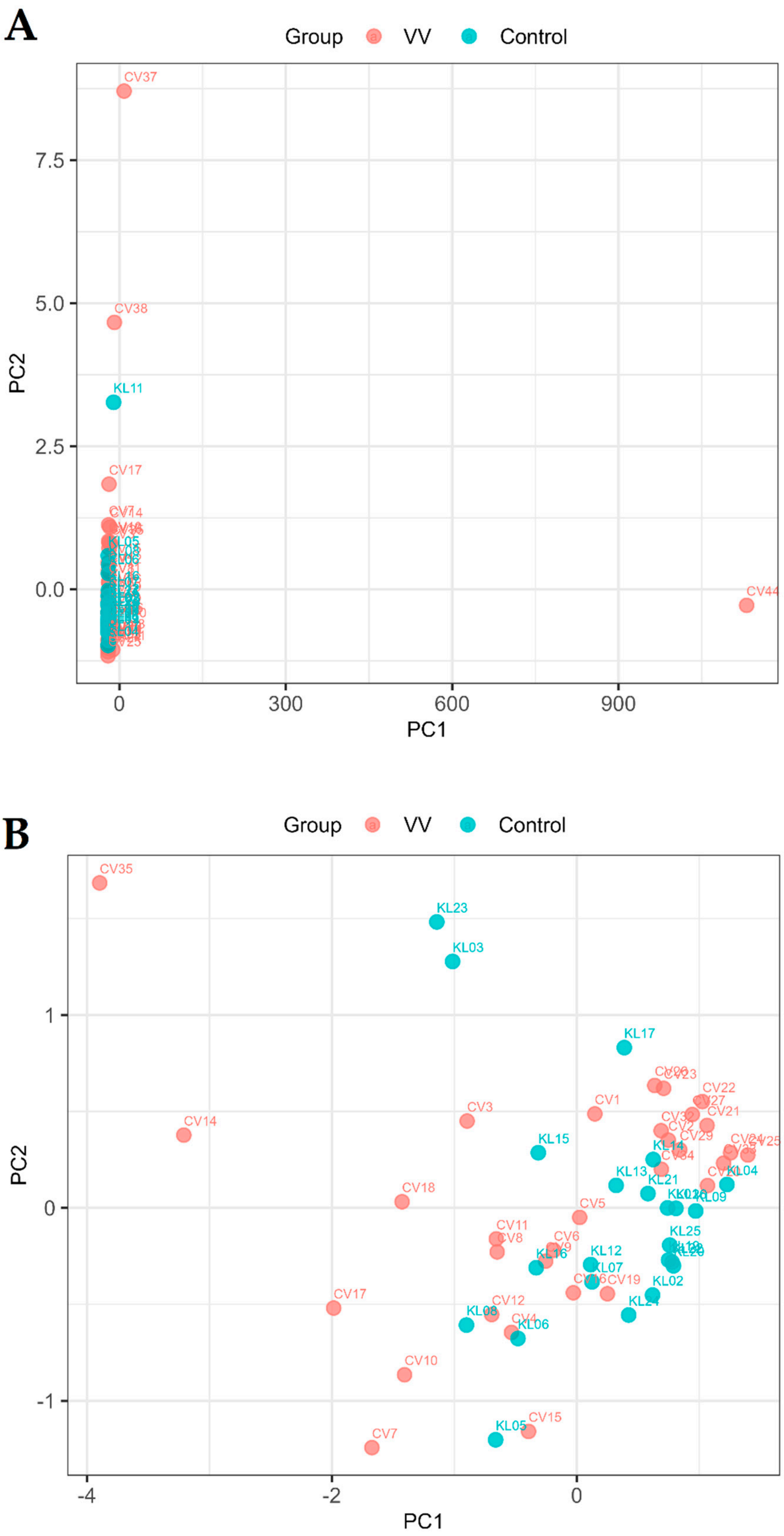

Figure S4. Spatial arrangement of samples using PCA components of gene expression data (2^{-dCt} values). (A) Results obtained before removing outlier samles and (B) after removing outlier samples.

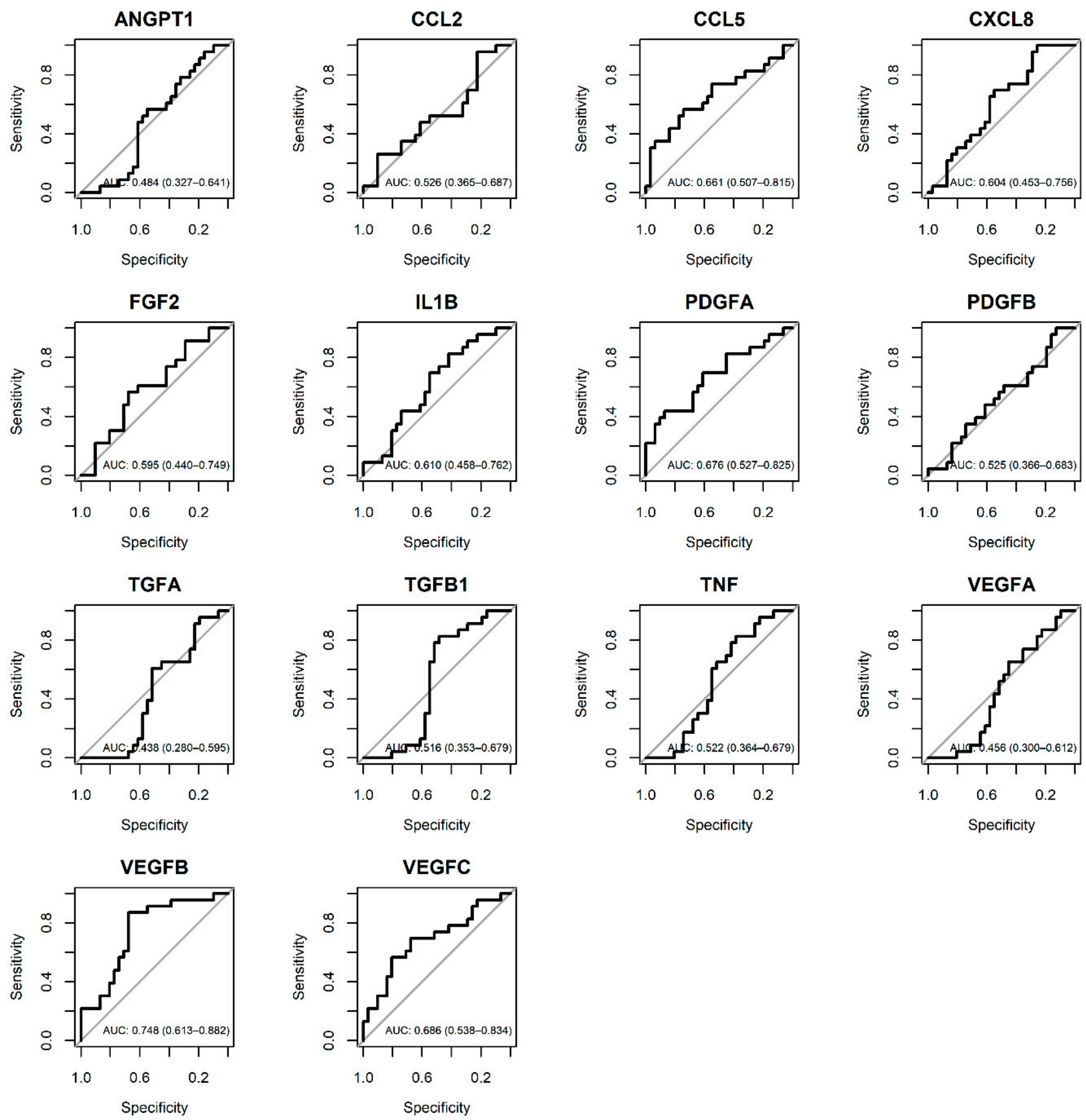

Figure S5. Results of Receiver Operating Characteristics (ROC) analysis performed for the studied genes.

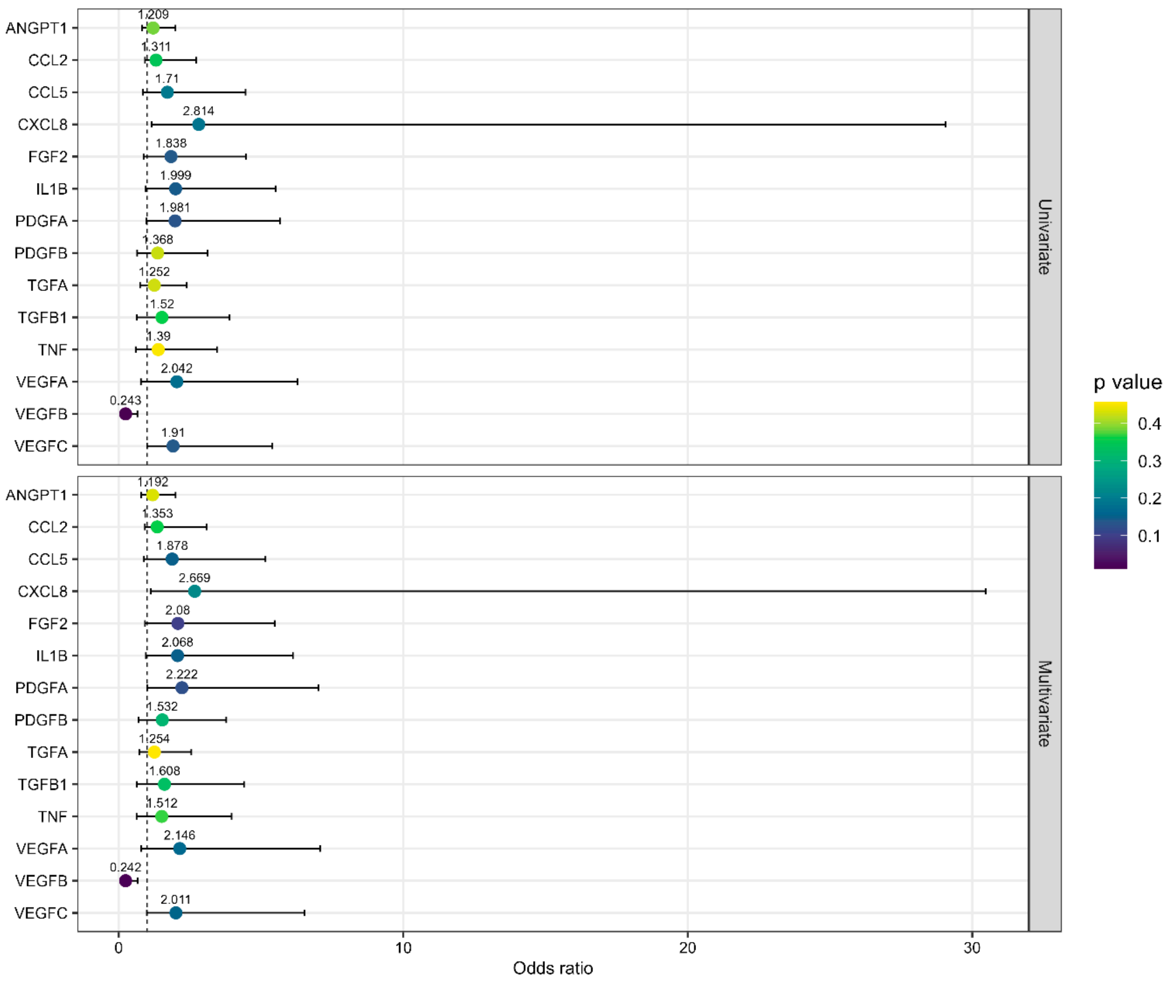

Figure S6. Odds ratios and their 95% confidence intervals and *p* values obtained in the univariate and multivariate logistic regression analysis (adjusted by age, sex, BMI, and smoking) for studied genes.

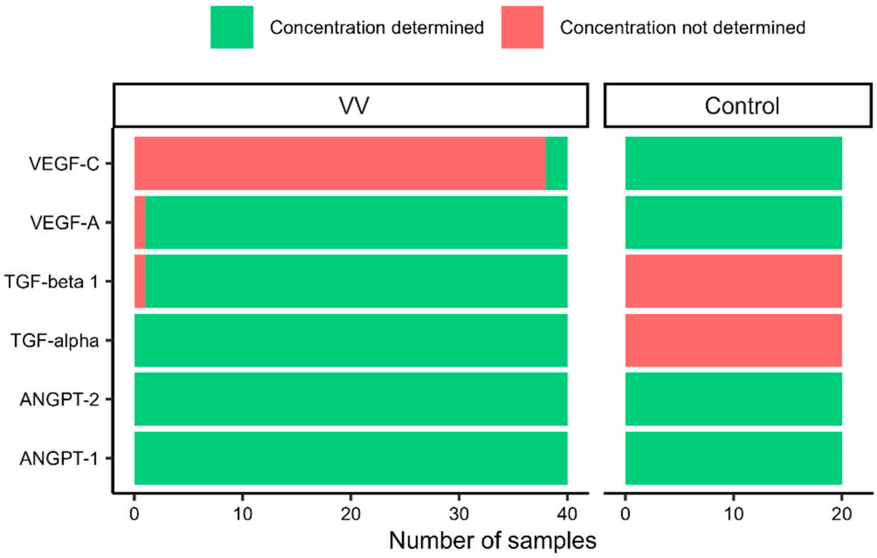

Figure S7. Amounts of samples in which plasma levels of analyzed proteins were determined or not.

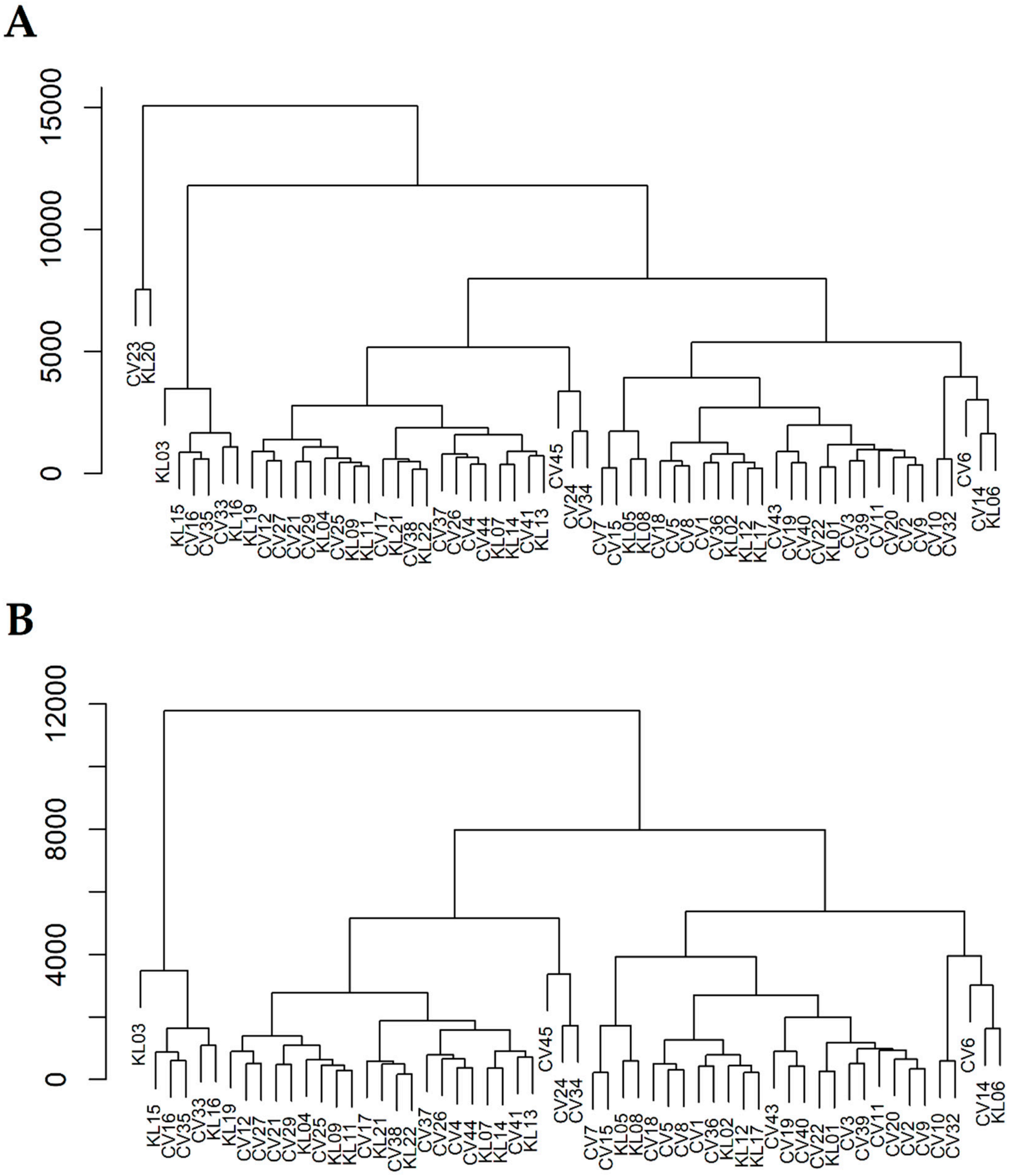

Figure S8. Hierarchical analysis performed for plasma levels of analyzed proteins in the studied samples using Euclidean distances. (A) Results obtained before and (B) after removing outlier samples.

A

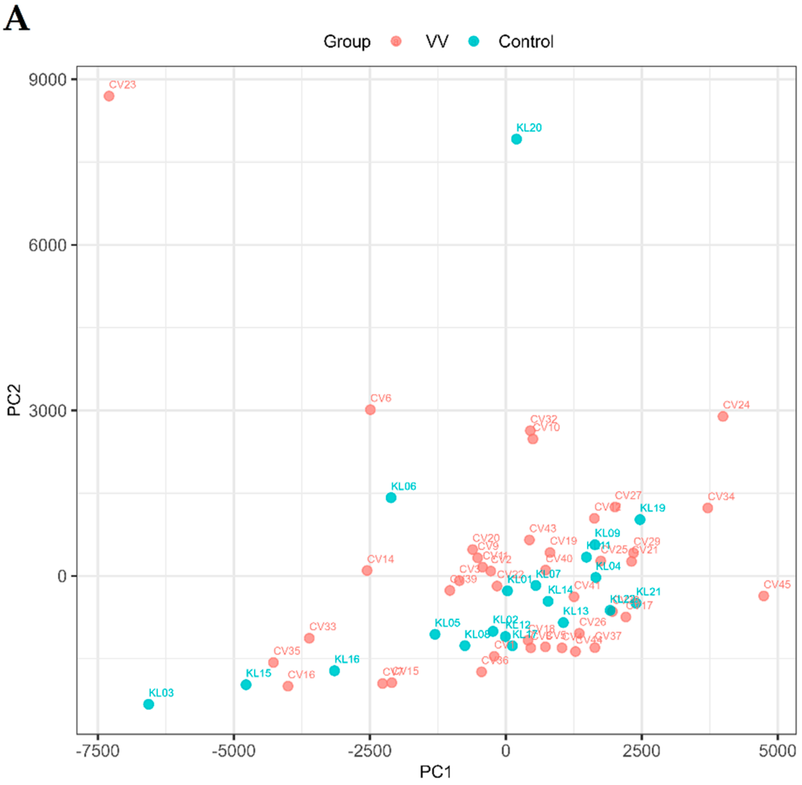

B

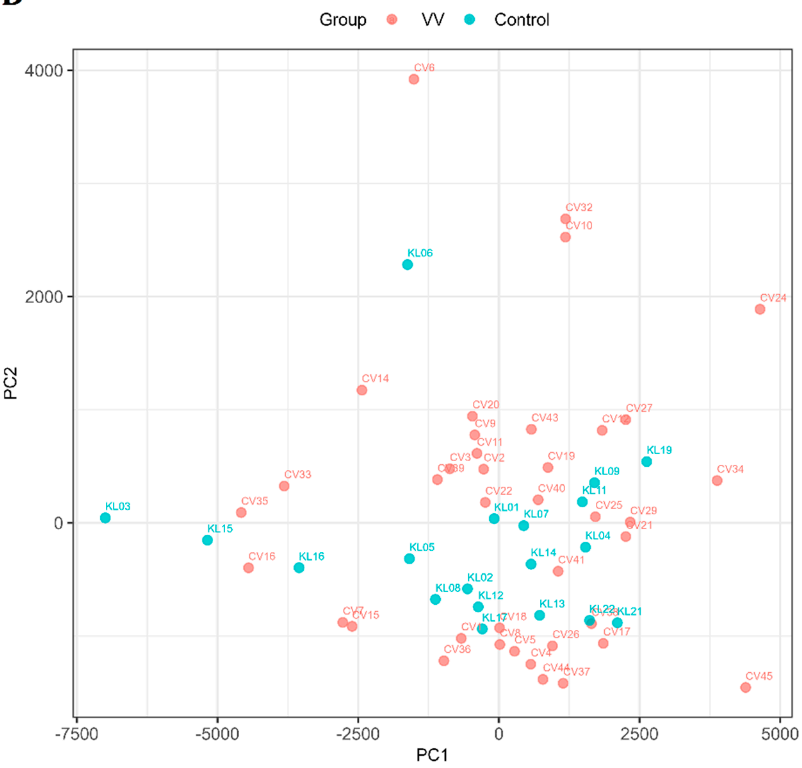

Figure S9. Spatial arrangement of samples using PCA components of protein plasma levels data. (A) Results obtained before and (B) after removing outlier samples.

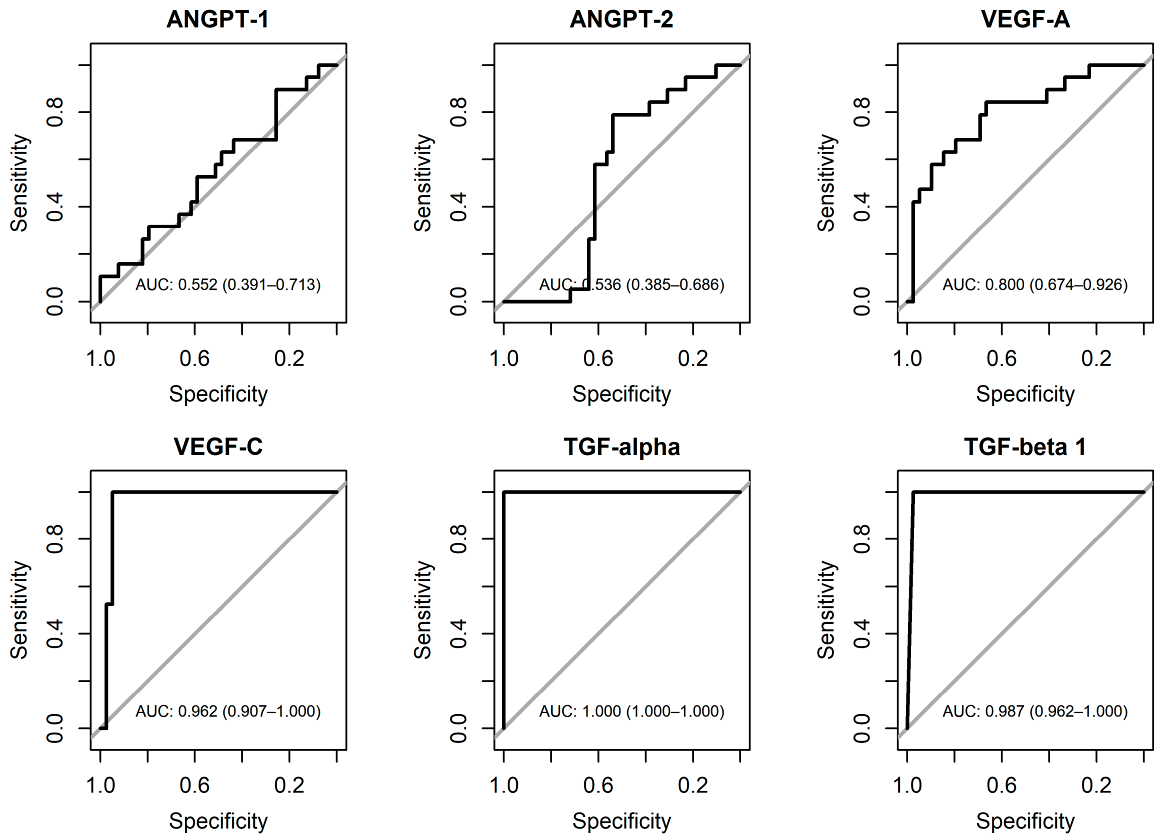

Figure S10. Results of Receiver Operating Characteristics (ROC) analysis performed for 6 analyzed proteins.

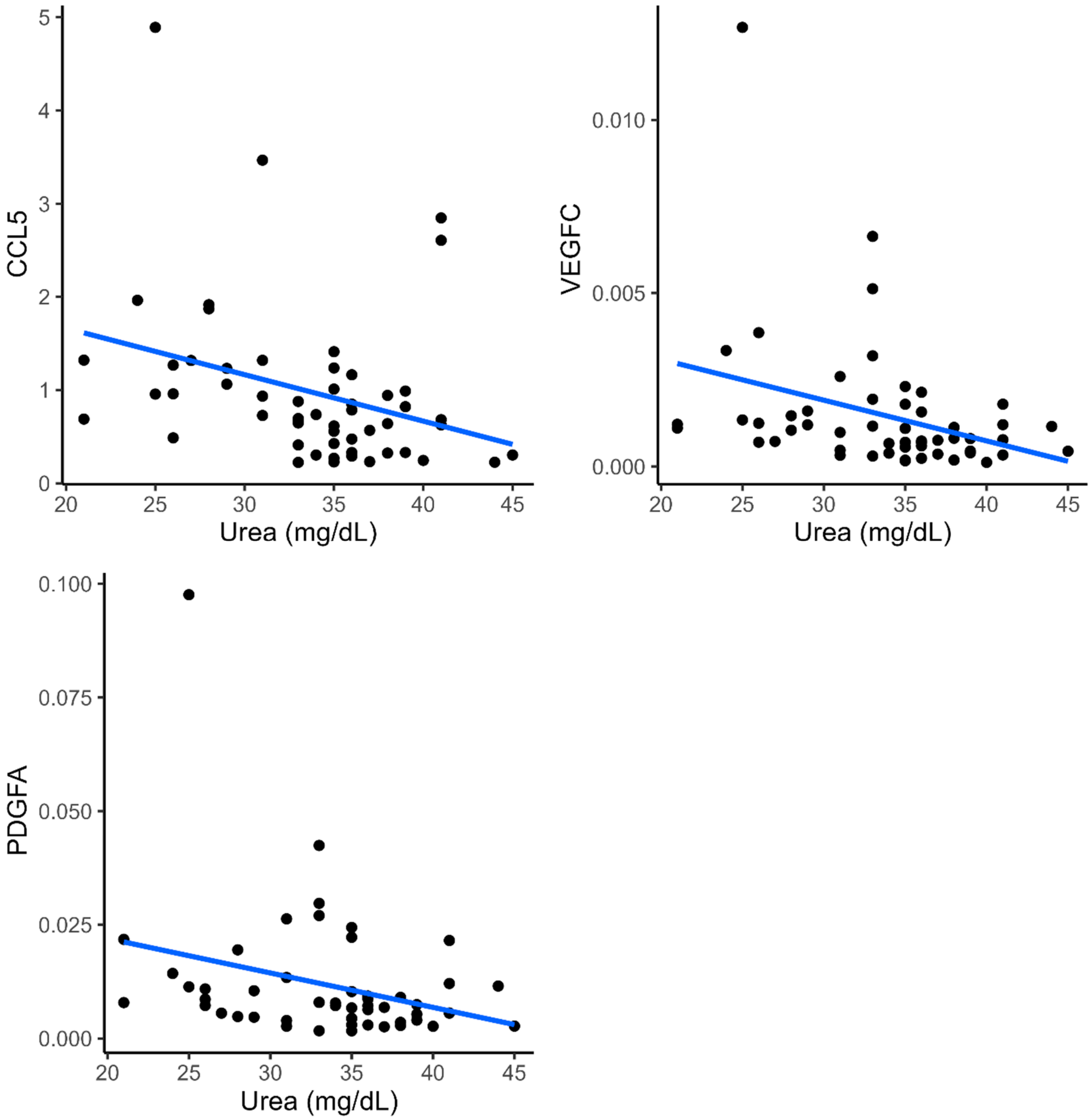

Figure S11. Statistically significant relationships between expression (2^{-dCt} values) of selected genes and clinical characteristics of the study subjects.

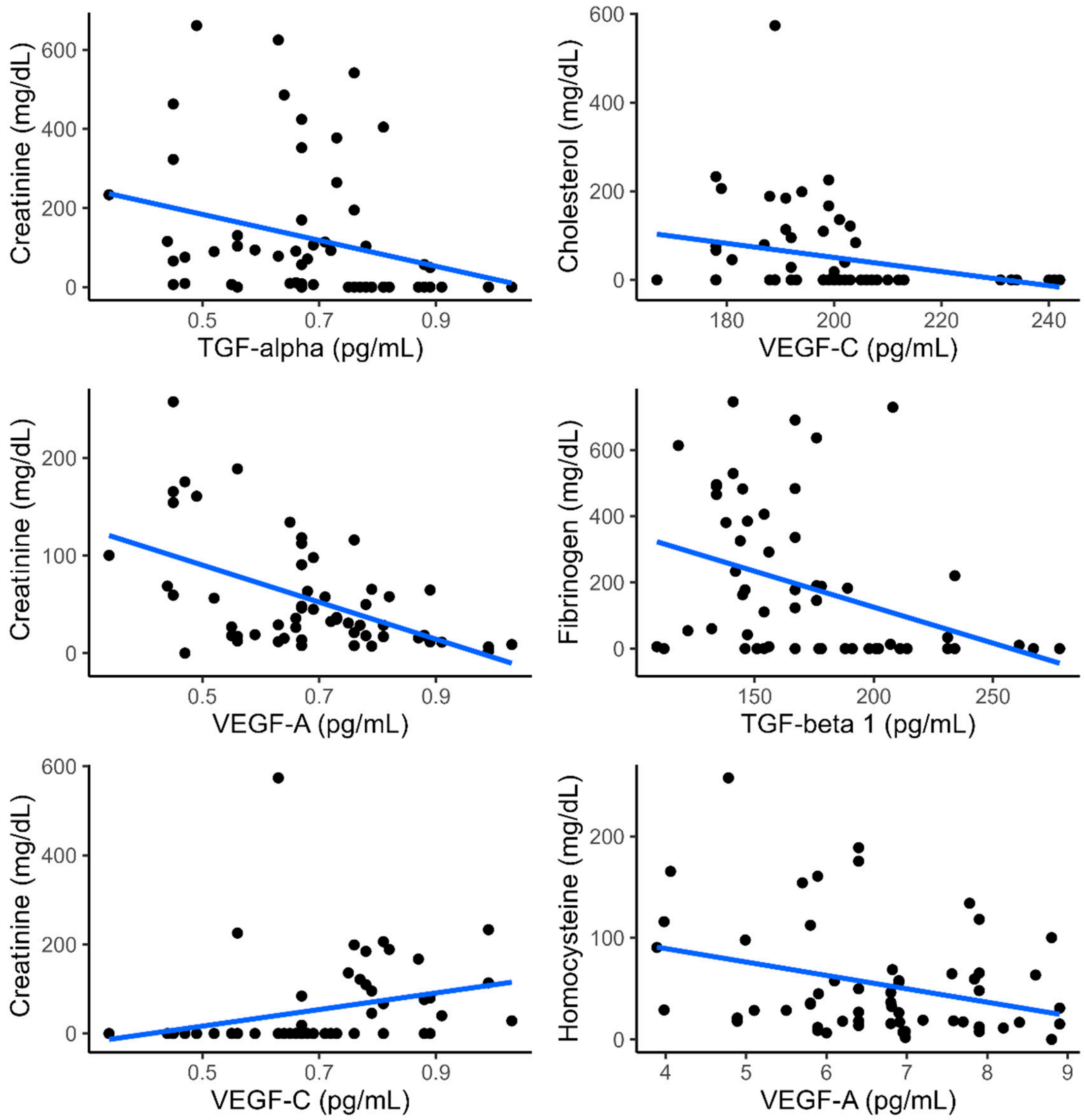

Figure S12. Statistically significant relationships between plasma levels of selected proteins and clinical characteristics of the study subjects.

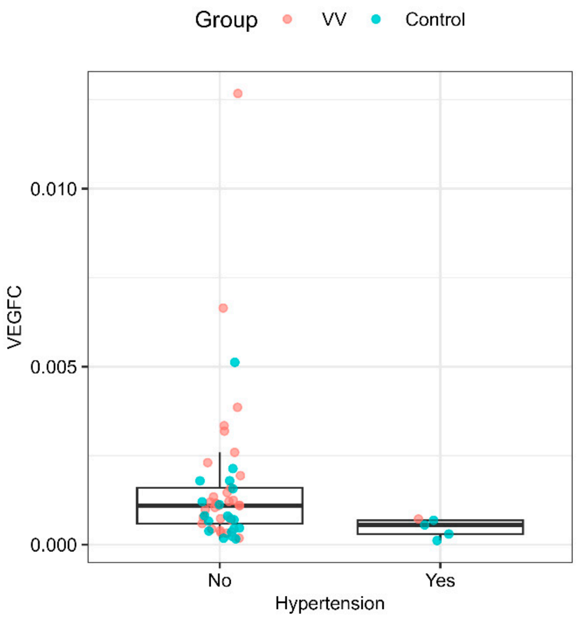

Figure S13. Distribution of *VEGFC* gene expression (2^{-dCt} values) in subjects with and without hypertension. Whiskers reach the most distant point in 1.5 interquartile range, boxes range between 25% and 75% quartile, horizontal lines inside boxes mark median value.

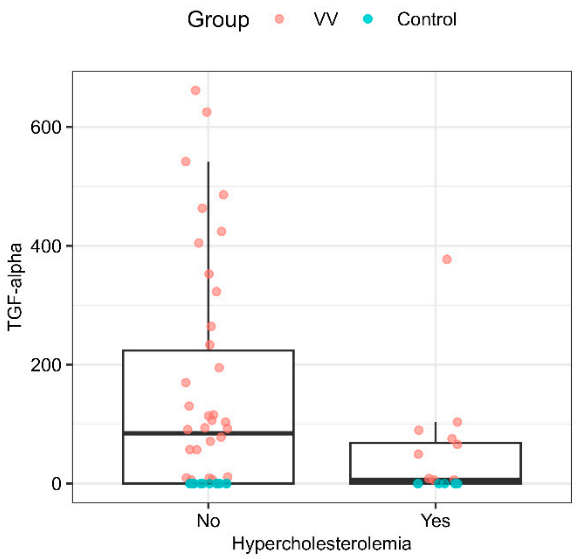

Figure S14. Plasma levels of TGF-alpha in subjects with and without hypercholesterolemia. Whiskers reach the most distant point in 1.5 interquartile range, boxes range between 25% and 75% quartile, horizontal lines inside boxes mark median value.
